# Does Facial Amimia Impact the Recognition of Facial Emotions? An EMG Study in Parkinson’s Disease

**DOI:** 10.1371/journal.pone.0160329

**Published:** 2016-07-28

**Authors:** Soizic Argaud, Sylvain Delplanque, Jean-François Houvenaghel, Manon Auffret, Joan Duprez, Marc Vérin, Didier Grandjean, Paul Sauleau

**Affiliations:** 1 Behavior and Basal Ganglia" research unit (EA4712), University of Rennes 1, Rennes, France; 2 Neuroscience of Emotion and Affective Dynamics laboratory, Department of Psychology and Educational Sciences, University of Geneva, Geneva, Switzerland; 3 Swiss Center for Affective Sciences, Campus Biotech, University of Geneva, Geneva, Switzerland; 4 Department of Neurology, Rennes University Hospital, Rennes, France; 5 Department of Neurophysiology, Rennes University Hospital, Rennes, France; Max Planck Institute for Human Cognitive and Brain Sciences, GERMANY

## Abstract

According to embodied simulation theory, understanding other people’s emotions is fostered by facial mimicry. However, studies assessing the effect of facial mimicry on the recognition of emotion are still controversial. In Parkinson’s disease (PD), one of the most distinctive clinical features is facial amimia, a reduction in facial expressiveness, but patients also show emotional disturbances. The present study used the pathological model of PD to examine the role of facial mimicry on emotion recognition by investigating EMG responses in PD patients during a facial emotion recognition task (anger, joy, neutral). Our results evidenced a significant decrease in facial mimicry for joy in PD, essentially linked to the absence of reaction of the *zygomaticus major* and the *orbicularis oculi* muscles in response to happy avatars, whereas facial mimicry for expressions of anger was relatively preserved. We also confirmed that PD patients were less accurate in recognizing positive and neutral facial expressions and highlighted a beneficial effect of facial mimicry on the recognition of emotion. We thus provide additional arguments for embodied simulation theory suggesting that facial mimicry is a potential lever for therapeutic actions in PD even if it seems not to be necessarily required in recognizing emotion as such.

## Introduction

Facial expression is a powerful non-verbal channel providing rapid essential clues for the perception of other people’s emotions, intentions and dispositions during social interactions. It constitutes a key component in daily social communication [1,2]. The processing of facial expressions normally contributes to behaviours that are appropriate to the emotion perceived and the situation and to the person with whom we are communicating. This ensures successful social interactions [3,4].

Facial mimicry is defined as the tendency to mimic the facial expressions of individuals with whom we are interacting, or at least to show congruent valence-related facial responses to the perceived expression. According to embodied simulation theory, facial mimicry could foster the understanding of emotion and/or facilitate the inferences and attributions about the mental states of others during social interactions. In the observer, the mirrored expression is linked to a central motor command and is thought to induce a tonic muscular change related to a central proprioceptive feedback; both phenomena are thought to help understand the emotion perceived from the clues displayed by the sender [5–7].

In this context, disturbed motor processing can lead to impairments in emotion recognition. This assumption is supported by experimental studies in which the facial feedback was inhibited or intensified by behavioural (holding a pen between the lips) or pharmacological (facial botulinum toxin injections) manipulations and by studies among people with facial expression disorders [8–11]. Nevertheless, these studies did not always yield conclusive results. For example, Bogart and Matsumoto [12] showed that performances among people with Moebius syndrome (a congenital condition resulting in facial paralysis) did not differ from those in the control group on a task assessing emotion recognition. The authors concluded that facial mimicry was not necessarily involved in the process of recognizing emotion. However, people with Moebius syndrome were able to perform as well as healthy people by using compensatory strategies, just as they do with their voices and bodies, to convey emotions [13]. Similarly, correlation analyses did not always confirm a relationship between mimicry and emotion recognition among healthy people [14–16].

Parkinson’s disease (PD) is another pathology affecting facial expression. One of the most distinctive clinical features of this neurodegenerative disorder is facial amimia: the reduction or loss of spontaneous facial movements and emotional facial expressions [17]. However, PD affects not only facial expressions but also body motion overall and vocal production. Thus, unlike Moebius syndrome people, PD patients may be less prone to compensating for their lack of facial expression by these alternative channels. In addition, PD should not be reduced to motor symptoms since research has shown that it is clearly also characterised by emotional dysfunctions. These disorders concern several components of emotion including subjective feeling, physiological arousal, emotion recognition and motor expression [18]. Thus, in the light of embodied simulation theory, PD patients may—at least in part—suffer from deficits in emotion recognition as a result of a reduced ability to mimic facial expressions [19–21]. Despite the fact that PD appears to be a useful model to address this issue, there has been no study on this topic to date. Such studies would be valuable given that the few investigations assessing the ability both to express and to recognize facial emotions in PD have evidenced positive correlations between impairments in facial expression and disturbances in emotion recognition [22,23].

In this context, the current study was designed to investigate both the recognition of facial emotion and facial mimicry in PD patients. We expected (1) to confirm the negative impact of PD in the recognition of emotion as reported in the literature, (2) to highlight a facial mimicry disturbance among PD patients and (3) to evidence a link between these two deficits.

## Materials and Methods

### Participants

Forty patients with Parkinson’s disease (PD) and 40 healthy controls (HC) took part in the study. All participants provided written informed consents and were informed of the confidential and anonymous aspect of their involvement in this study which was approved by the ethical committee on human experimentation of the Rennes University Hospital, France. All clinical investigation has been conducted according to the principles expressed in the Declaration of Helsinki. Each participant underwent a neuropsychological and psychiatric interview in order to control for any potential bias factors. They were required to obtain a minimum standard score of 5 on the Matrix Reasoning subtest from the Wechsler Adult Intelligence Scale [24] and to report normal or corrected-to-normal visual acuity. Concerning visuospatial ability, cut-off scores used for patients inclusion were as follows: 15 on the shape detection screening subtest from the Visual and Object Space Perception battery (VOSP), 18 on the position discrimination VOSP subtest and 7 on the number location VOSP subtest [25]. Non-emotional face recognition abilities were checked on the Benton unfamiliar-face matching test [26]; cut-off score for inclusion = 39. No participant had received injections of dermal filling or dermo-contraction agents in the facial muscles; none reported any history of neurological or psychiatric disease (except for PD) or drug/alcohol abuse. To ascertain the absence of apathy, a score higher than -7 on the Lille Apathy Rating Scale (LARS) was required for each patient [27]. In addition, the participants completed the State-Trait Anxiety Inventory (STAI; [28]).

Disease severity was rated on the Unified Parkinson’s Disease Rating Scale motor part (UPDRS III; [29] and the Hoehn and Yahr disability scale [30], both under dopamine replacement therapy (ON DRT) and during a temporary withdrawal from treatment (OFF DRT). For the OFF DRT evaluation, the patients were asked not to take their medication as from the night before the assessment. A levodopa-equivalent daily dose (LEDD) was calculated for each patient [31]. The patients were on their usual medication during the experiment.

Finally, since caring for someone with PD is associated with socio-emotional distress [32,33], caregivers including spouses were not included as HC.

### Experimental design

**Fig 1:**
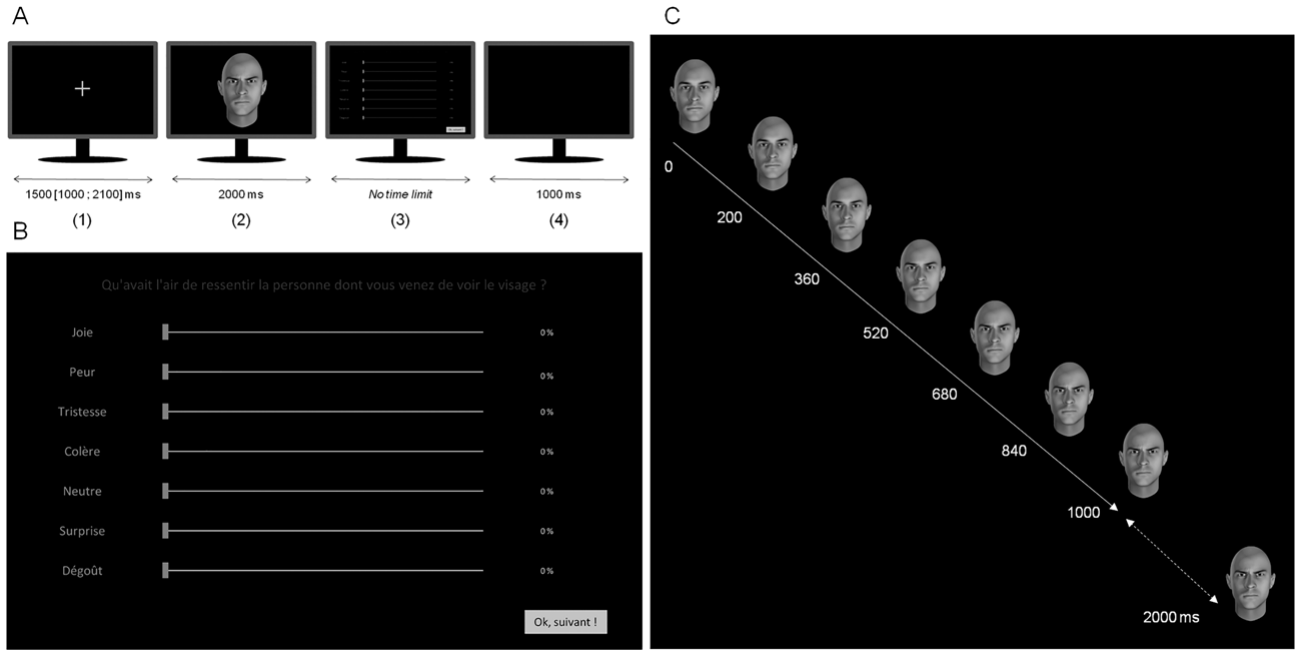
Facial emotion recognition task and stimulus material. Timing (A) and response interface (B) of the experiment. Example of facial expression display time (C). The emotional expression unfolded from a neutral state (0) to its emotional peak in 1000 ms (solid line) and stayed at the emotional apex for an additional 1000 ms (dotted line).

For each trial, a dynamic avatar appeared on a black screen for 2000 ms (Fig 1). Then, the participants assessed the emotions portrayed and their intensities on seven visual analogue scales (VAS) labelled joy, sadness, fear, anger, disgust, surprise and neutral. The VAS ranged from 0 to 100%. We used a total of 36 stimuli, presented pseudo-randomly and divided in 3 blocks of 12. In the whole experiment, each participant was exposed to the 36 stimuli with 12 different avatars portrayed 3 different expressions (anger, joy, neutral). The stimuli consisted in naturally coloured Caucasian avatars (6 women/6 men). For all stimuli, we used FACSGen [34,35] to generate videos clips in which the emotional expression unfolded from a neutral state to its emotional peak in 1000 ms and remained at this level for another 1000 ms. See S1 Appendix for a more detailed description of the experimental material.

### EMG recordings

Bipolar measures of facial EMG activity were performed using Ag/AgCl electrodes, 3 mm in diameter, filled with a highly conductive water electrolyte gel (F-E9M SAFELEAD^™^ model and EC60 Signalgel^®^, Grass Technologies). According to the guidelines [36], six electrodes were positioned over the left zygomaticus (*zygomaticus major)*, orbicularis (*orbicularis oculi*) and corrugator (*corrugator supercilii)* muscle regions and one reference electrode (15x20 mm, Ambu^®^ Neuroline 700) was attached to the forehead. In order to avoid any potential participant bias, we recorded facial muscle activity in participants being not aware that this was the real purpose of the electrodes. The use of facial electrodes was explained by the need for recording sweat gland activity. The only instruction given to the participants was to assess the emotion portrayed by the avatar using the VAS. No reference to muscle activity or facial movements was given at any time of the experiment. To further confirm that the participants were not aware of the objective of the study, we interviewed them after the experiment. None of the participants reported that they had figured out our purpose or that they had focused on facial movements. The EMG raw signal was recorded with a g.BSamp biosignal amplifier (g.tec), digitized using a 16-bit analog-to-digital converter and stored with a sampling frequency of 1000 Hz. Offline, it was filtered with a 40–200 Hz band pass filter, rectified and smoothed with a sliding average (window size = 200 ms). PowerLab 16/35 hardware and LabChart^®^ Pro Software version 7.3.7 (ADInstrument) were used for EMG data acquisition. After a visual examination of the EMG signals recorded from one second before stimulus onset to stimulus offset, trials in which the EMG amplitude exceeded 30 μV were removed in order to reject any remaining artefacts (3.04% of trials deleted).

### Data analysis

Five trials out of 2880 were excluded from the analyses because no response was recorded on the VAS.

#### Facial emotion recognition

First, the performances on the emotion recognition task were exploited in terms of emotion decoding accuracy. An expression was considered as accurately identified when the emotion receiving the highest intensity rating on the VAS corresponded to the emotion displayed (target emotion). The accurately identified expressions were coded as 1; misidentified expressions were coded as 0. Then, in order to determine the nature of the confounding emotions in case of misidentified expressions, confusion percentage was calculated for each non-target emotion; emotions that did not corresponded to the relevant stimulus (number of times each non-target emotion received the highest intensity rating on the VAS instead of the target emotion on the total number of trials).

#### Facial EMG responses

For each trial, the second before stimulus onset was considered as baseline. To examine the temporal profiles of facial reactions, the EMG amplitudes were averaged across the sequential 100 ms intervals of stimulus exposure and expressed as a relative percentage of the mean amplitude for baseline. As we expected to highlight a dynamic pattern of facial reactions to emotions as already reported in the literature, facial EMG responses were calculated as previously but on sequential 500 ms periods of stimulus exposure (0–500; 500–1000; 1000–1500 and 1500–2000 ms) to examine the effect of the patients’ clinical characteristics as well as to assess the relationship between emotion recognition and facial responses (see S1 Fig).

### Statistical analysis

Data management and statistical analyses were performed using R 3.2.0 [37]. The significance threshold was set at *α* = 0.05 except when it was adjusted for multiple comparisons.

The experimental fixed effects on decoding accuracy (group and emotion) and on EMG responses (group, emotion, muscle and interval) were tested by fitting logistic and linear mixed models respectively, with random intercepts for both participants and avatars (“glmer” and “lmer” functions in the “lme4” package, (g)lmer{lme4}; [38]). In order to control for bias factors, the sociodemographic and neuropsychological variables, as well as their interaction effects with the group factor, were added to these models as fixed factors. Then, analyses of variance (type II Wald Chi-square tests, Anova{car}) were computed and only the potential bias factors with significant effects were retained in the models in order to increase their statistical power. In the case of significant effects, contrasts were tested (testInteractions{phia}) using the Bonferroni adjustment method for *p* values [39,40]. The fixed effects of group and non-target emotion on confusion percentage were tested similarly by fitting linear mixed models with random intercepts for participants for each level of emotion factor. In order to test whether muscles exhibited higher activity at baseline in the PD patients, analysis of variance was computed on mean EMG amplitudes measured at baseline with group, muscle and emotion as fixed factors and random intercepts for both participants and avatars. As previously, contrasts were tested using the Bonferroni adjustment method for *p* values in case of significant effects. The impact of medication and other clinical characteristics such as disease severity on decoding accuracy and EMG responses was tested by computing analyses of variance for fitted logistic and linear mixed models with disease duration, the worst affected side, LEDD, Hoehn and Yahr stages and UPDRS III scores (ON and OFF DRT) as fixed factors in addition to the experimental factors (emotion and muscle). For the effects of these clinical characteristics on EMG responses, analyses were conducted on the standardized EMG responses calculated across sequential 500 ms periods of stimulus exposure for each level of muscle, emotion and period factors. Finally, to examine the relationship between emotion recognition and facial mimicry, the fixed effects of the group, the standardized EMG responses of the three recorded muscles calculated on sequential 500 ms periods and their interaction with the group on decoding accuracy were tested by computing analyses of variance for logistic mixed models fitted for each level of emotion and period factors.

## Results

### Characteristics of the groups and confounding factors

The group characteristics are shown in Table 1. For details about the patients’ medication, refer to S1 Table. All the sociodemographic and neuropsychological factors involving potential bias (age, gender, state and trait anxiety levels, scores on the Matrix and on the Benton tests) were added to the statistical models fitted to assess the effects of experimental factors on decoding accuracy and on EMG responses. Only a negative impact of age on decoding accuracy was significant (*χ²* = 8.87, *df* = 1, *p* = 0.003).

**Table 1:** Characteristics of the groups with mean ± standard deviation [range] and statistics.

	N	Healthy controls	PD patients		*(df)* F	*p* value
			ON DRT	OFF DRT		
Gender (F/M)	40/40	20/20	20/20	-	-	-
Age (years)	40/40	62.2 ± 7.8 [43; 75]	61.2 ± 9.6 [42; 79]	-	*(1*,*78)* 0.29	0.59
State anxiety [20; 80]	40/38	27.7 ± 7.3 [20; 48]	31.4 ± 6.6 [20; 47]	-	*(1*,*76)* 5.61	0.02
Trait anxiety [20; 80]	40/35	35.2 ± 6.5 [20; 50]	39.1 ± 8 [24; 58]	-	*(1*,*73)* 5.49	0.02
Matrix [1; 19]	36/37	13.1 ± 2.4 [7; 18]	9.9 ± 2.5 [5; 15]	-	*(1*,*71)* 31.63	<0.001
Benton (/54)	39/38	46.9 ± 3.5 [39; 52]	45 ± 3.2 [40; 53]	-	*(1*,*75)* 5.96	0.017
VOSP SDS (/20)	-/38	-	19.7 ± 0.6 [18; 20]	-	-	-
VOSP PD (/20)	-/38	-	19.7 ± 0.6 [18; 20]	-	-	-
VOSP NL (/10)	-/38	-	9.1 ± 0.9 [7; 10]	-	-	-
LARS [-15; 15]	-/37	-	-11.8 ± 2.6 [-15; -7]	-	-	-
Disease duration (years)	-/40	-	9.7 ± 5.3 [1; 20]	-	-	-
Worst affected side (L/R)	-/40	-	17/23	-	-	-
LEDD (mg/day)	-/40	-	1043.2 ± 454.2 [250; 2355]	-	-	-
UPDRS III^1^ (/108)	37/32	-	11.9 ± 8.9 [1; 33]	30.5 ± 12.6 [14; 62.5]	*(1*,*67)* 50.31	<0.001
Hoehn & Yahr^1^ (/5)	38/39	-	1.2 ± 0.7 [0; 3]	2.1 ± 1 [1; 5]	*(1*,*75)* 23.43	<0.001

N = data available for each group: healthy participants/PD patients.

^1^number of patients assessed during the ON and OFF DRT states: ON/OFF. VOSP SDS, PD and ND = Shape Detection Screening, Position Discrimination and Number Location subtests from the Visual and Object Space Perception battery (VOSP). *df* = degrees of freedom. For the participants who could not be assessed on the Matrix test, a minimum score of 130 on the Mattis Dementia Rating Scale [41] was nonetheless used to ascertain the absence of cognitive dysfunctions.

### Facial emotion decoding accuracy

**Fig 2:**
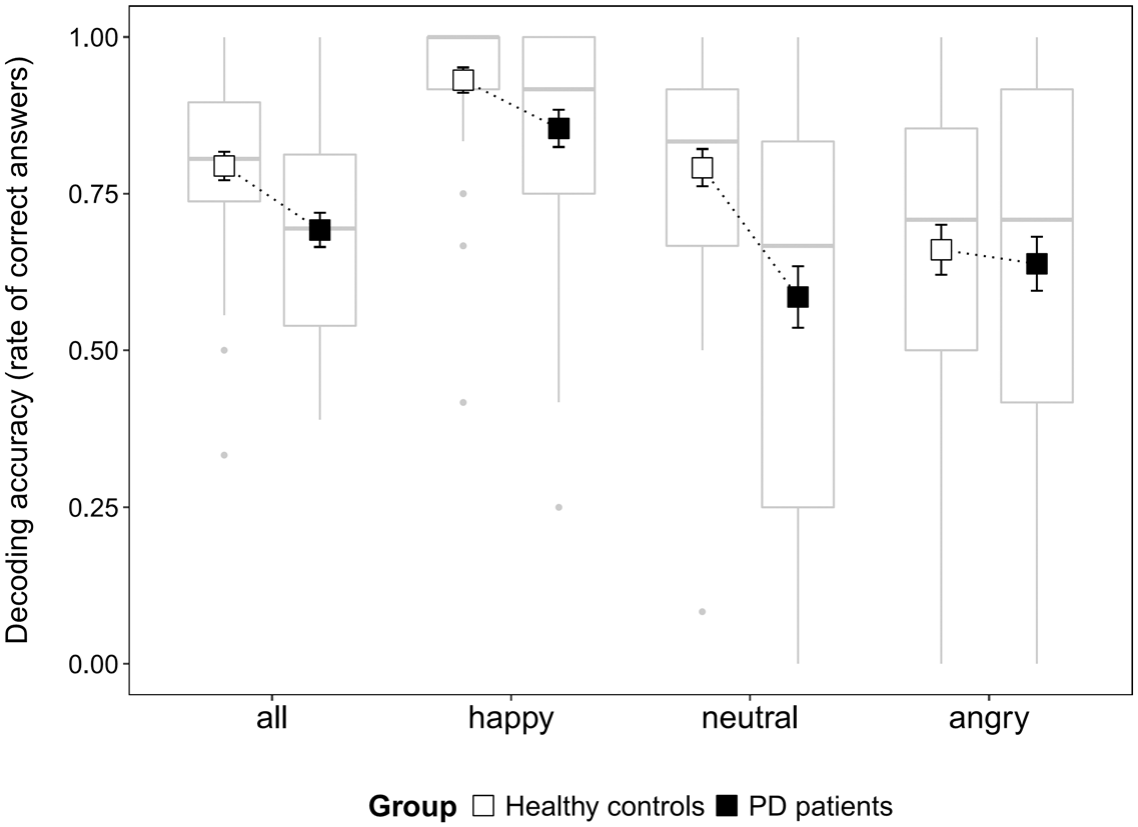
Decoding accuracy scores of the healthy controls and the PD patients whatever the emotion displayed (all) and as a function of emotion. Mean ± standard error and boxplot.

Decoding accuracy varied across experimental conditions (Fig 2). The decoding accuracy scores of the PD patients were overall significantly lower than those of the HC (*χ²* = 13.34, *df* = 1, *p*<0.001). A significant group x emotion interaction effect (*χ²* = 21.05, *df* = 2, *p*<0.001) showed that the difference between groups was interacting with the emotion factor. The decoding accuracy scores of the PD patients were significantly lower than those of the HC for happy (*χ²* = 7.07, *df* = 1, *p* = 0.024) and neutral avatars (*χ²* = 14.28, *df* = 1, *p*<0.001) but not for angry faces (*χ²* = 0.2, *df* = 1, *p* = 0.99). In the PD patients, performances tended to increase with the LEDD (*χ²* = 2.74, *df* = 1, *p* = 0.098). No effect was found for disease duration or severity measured by the Hoehn and Yahr stages or the UPDRS III scores (ON and OFF DRT; all *p*>0.1).

### Confounding emotions

**Fig 3:**
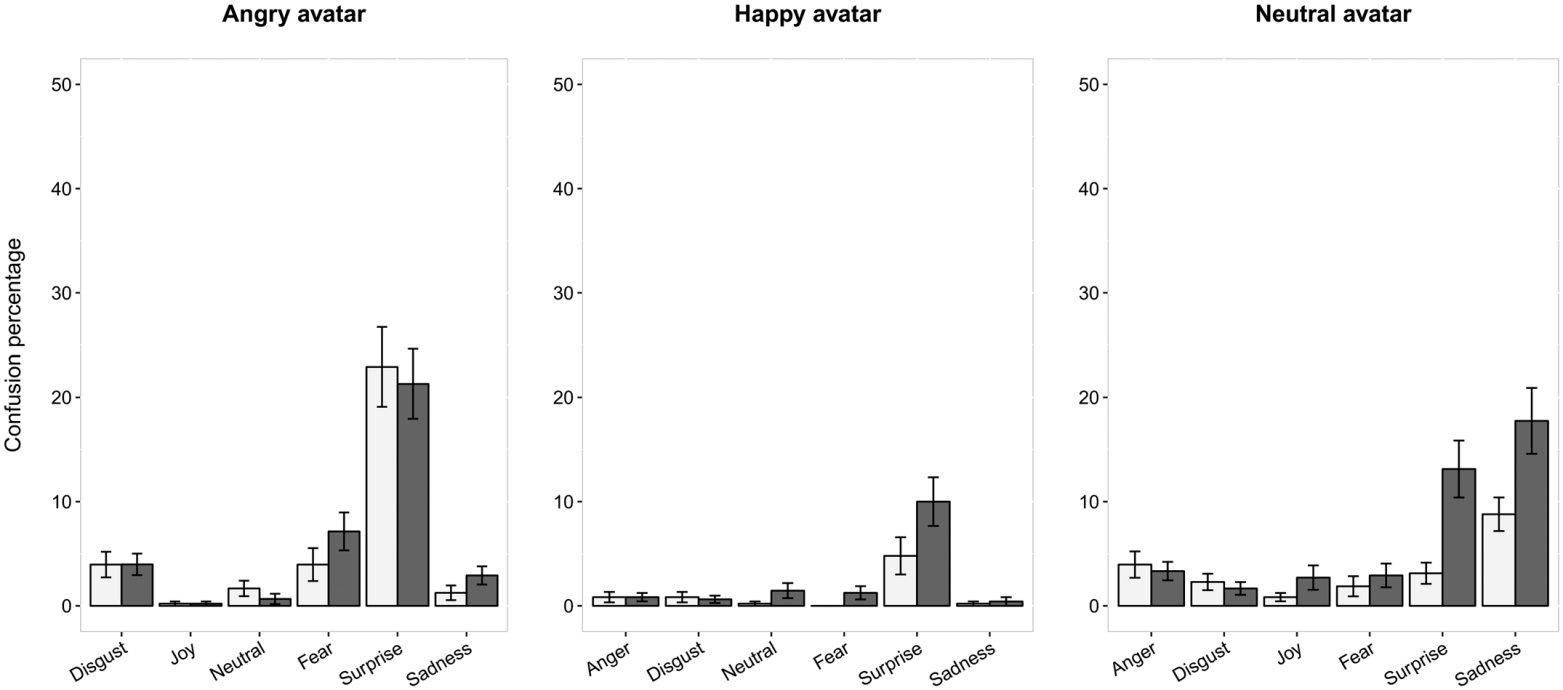
Emotional nature of the misidentified expressions as a function of group and emotion displayed. Mean ± standard errors. Confusion percentage among the HC are shown in white; confusion percentage among the PD patients are shown in grey.

When the emotional nature of the error made was examined, analyses showed that the PD patients provided globally a similar pattern of confusion than the HC. However, quantitative and qualitative differences appeared for some aspects (Fig 3). For angry avatars, the confounding emotion was mostly surprise, then fear and disgust, in both groups. For happy avatars, the confounding emotion was quasi-systematically surprise in both groups. For neutral avatars, the confounding emotion was sadness in both groups, then surprise among the PD patients. The group x non-target emotion interactions were statistically significant for happy (χ² = 12.06, df = 5, p = 0.034) and neutral (*χ²* = 25.21, *df* = 5, *p*<0.001) avatars but not for angry avatars (*χ²* = 2.6, *df* = 5, *p* = 0.76). Surprise was more often selected by the PD patients than the HC for happy avatars (*χ²* = 15.33, *df* = 1, *p*<0.001) and neutral avatars (*χ²* = 21.25, *df* = 1, *p*<0.001). For neutral avatars, the PD patients also selected more often sadness than the HC did (*χ²* = 17.06, *df* = 1, *p*<0.001).

### Facial reactions

**Fig 4:**
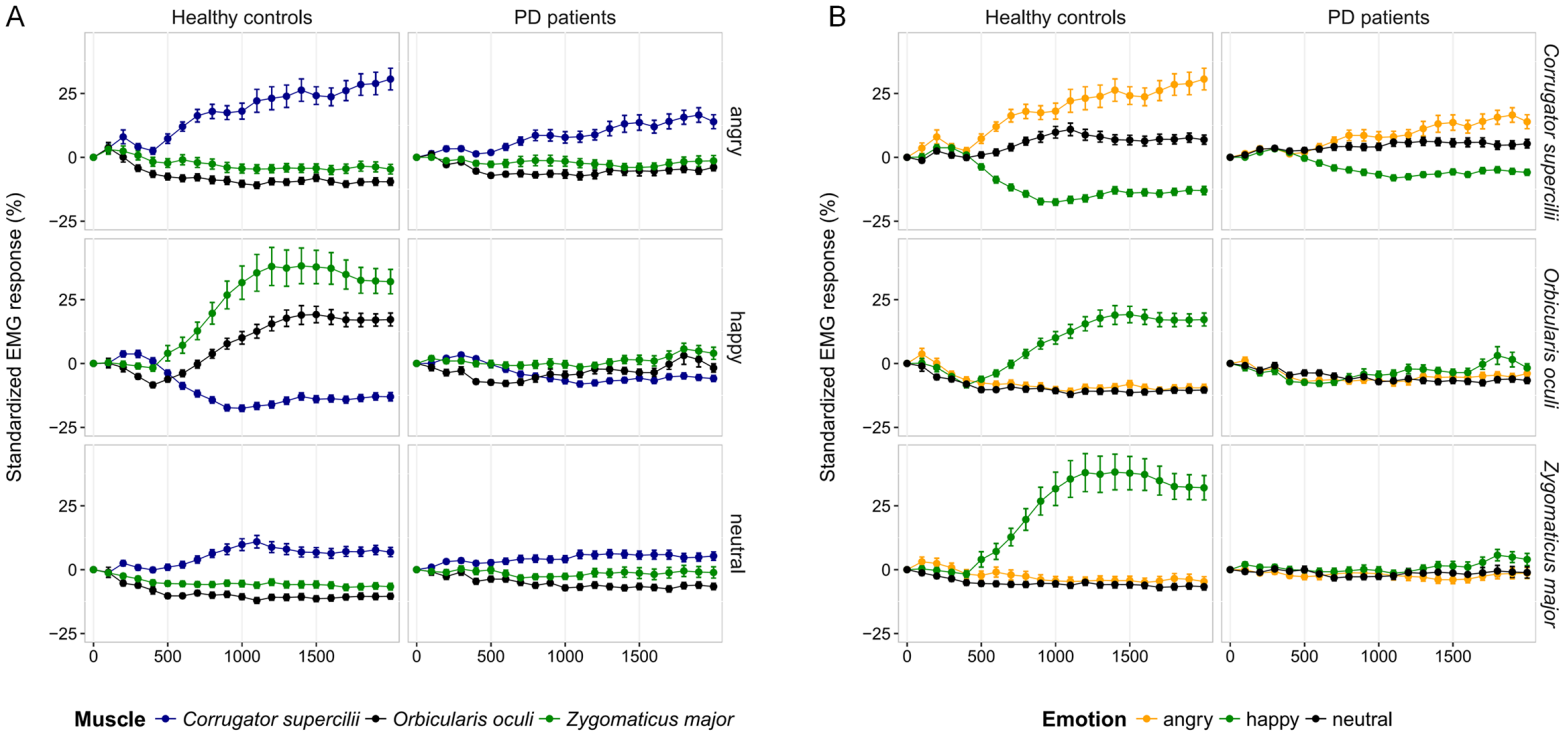
EMG responses (relative to baseline) recorded on sequential 100 ms intervals of stimulus exposure according to muscle, group and emotion factors. Mean ± standard error. (A) emotion-specific variations for each recorded muscle; (B) muscle-specific variations in response to the emotion displayed.

A statistically significant group x emotion x muscle x interval interaction effect was found (*χ²* = 431.65, *df* = 76, *p*<0.001). In the HC, comparisons across emotions evidenced specific variations in response to the emotion for each muscle (Fig 4A, S2 Table). From 400 ms after stimulus onset, corrugator activity decreased in response to expressions of joy whereas it increased in response to angry faces and showed an intermediate pattern in response to neutral expressions. Conversely, from 500 ms after stimulus onset, zygomaticus activity increased in response to expressions of joy whereas it remained quite stable or decreased slightly in response to angry or neutral avatars. The same applied to the orbicularis muscle from 700 ms after stimulus onset. Whatever the recording interval, the variations of these two muscles in response to angry avatars were not different from those observed in response to neutral expressions. In the PD patients, comparisons across emotions evidenced specific variations in response to the emotion for the corrugator muscle alone (Fig 4A, S3 Table). From 700 ms after stimulus onset, corrugator activity decreased in response to expressions of joy, increased slightly in response to angry faces and showed an intermediate pattern in response to neutral expressions (there was no significant difference between neutral and angry avatars except from 1700 to 1900 ms). Among the PD patients, the responses of the zygomaticus and the orbicularis muscles did not vary across emotions whatever the interval. Comparisons between muscle responses confirmed these specific variations of muscular activity observed in response to emotions (Fig 4B; see S2 Appendix, S4 and S5 Tables).

The analyses also evidenced a statistically significant group x emotion x muscle interaction effect (*χ²* = 1127.07, *df* = 4, *p*<0.001). Overall, the increased activity of the corrugator muscle in response to angry avatars was lower in the patients than in the HC (*χ²* = 14.89, *df* = 1, *p* = 0.001). More precisely, corrugator activity in response to angry avatars differed between the groups from 1000 to 1200 ms and from 1900 to 2000 ms after stimulus onset (Table 2). Likewise, the overall increased activity in both the zygomaticus (*χ²* = 78.96, *df* = 1, *p*<0.001) and the orbicularis (*χ²* = 23.29, *df* = 1, *p*<0.001) muscles in response to happy avatars were greater in the HC than in the patients for whom these responses were almost non-existent. These significant inter-group differences appeared from 700 ms after stimulus onset for the zygomaticus muscle and from 900 ms for the orbicularis muscle (Table 2). In addition, the overall decrease in corrugator activity in response to happy expressions tended to be lower in the patients compared to the HC (*χ²* = 6.65, *df* = 1, *p* = 0.089).

**Table 2:** Inter-groups comparisons of EMG responses recorded on sequential 100 ms intervals of stimulus exposure.

Interval	CORRU—Angry	ZYGO—Happy	ORBI—Happy
0–100	(0.3) ns	(0.2) ns	(0.2) ns
100–200	(1.5) ns	(0.1) ns	(0.3) ns
200–300	(0.03) ns	(0.3) ns	(0.4) ns
300–400	(0.08) ns	(0.2) ns	(0.1) ns
400–500	(2) ns	(1.3) ns	(0.1) ns
500–600	(4.4) ns	(4.5) ns	(1.2) ns
600–700	(7) ns	(13.15) = 0.052	(3.6) ns
700–800	(6.2) ns	**(28.7) <0.001**	(6.4) ns
800–900	(5.6) ns	**(50.7) <0.001**	(10.3) ns
900–1000	(7.4) ns	**(73.2) <0.001**	**(15.6) <0.05**
1000–1100	**(14) <0.05**	**(97.3) <0.001**	**(19.9) <0.005**
1100–1200	**(14.5) <0.05**	**(107.1) <0.001**	**(22.5) <0.001**
1200–1300	(11.4) ns	**(97) <0.001**	**(28.7) <0.001**
1300–1400	(12.4) = 0.079	**(95.9) <0.001**	**(34.2) <0.001**
1400–1500	(7.8) ns	**(94.3) <0.001**	**(37.1) <0.001**
1500–1600	(9.7) ns	**(93.7) <0.001**	**(34) <0.001**
1600–1700	(10.3) ns	**(73.1) <0.001**	**(21.8) <0.005**
1700–1800	(11.7) ns	**(51.5) <0.001**	**(13.9) <0.05**
1800–1900	(10.8) ns	**(53.6) <0.001**	**(17.3) <0.01**
1900–2000	**(19.8) <0.005**	**(56.1) <0.001**	**(25.8) <0.001**

Only the inter-groups comparisons of EMG responses recorded for the corrugator muscle in response to angry avatars (CORRU—Angry), the zygomaticus in response to happy avatars (ZYGO—Happy) and the orbicularis in response to happy avatars (ORBI—Happy) are shown here. No other comparisons were significant (all *p*>0.1). Test statistics (*χ^2^*) are shown in brackets. Figures in bold denote statistically significant differences (*p* value<0.05). ns = non statistically significant = *p* value>0.1

Regarding the mean EMG amplitudes measured at baseline, analysis showed a statistically significant group effect (*χ²* = 5.49, *df* = 1, *p* = 0.019) as well as a statistically significant group x muscle interaction effect (*χ²* = 124.78, *df* = 2, *p*<0.001). The mean EMG amplitudes measured at baseline were higher among the PD patients compared to the HC (mean ± standard error: PD patients = 5.25 ± 0.08 μV; HC = 3.91 ± 0.05 μV) but this effect was carried by the zygomaticus activity (PD = 6.02 ± 0.18 *vs*. HC = 3.69 ± 0.1 μV; *χ²* = 16.38, *df* = 1, *p*<0.001) as no statistically significant group difference emerged for the corrugator and orbicularis activities (corrugator: PD = 5.19 ± 0.1 *vs*. HC = 4.49 ± 0.09 μV; *χ²* = 1.16, *df* = 1, *p =* 0.85 and orbicularis: PD = 4.56 ± 0.12 *vs*. HC = 3.54 ± 0.05 μV; *χ²* = 3.65, *df* = 1, *p =* 0.17).

Finally, none of the clinical characteristics of the patients (disease duration, worst affected side, LEDD, Hoehn and Yahr stages and UPDRS III scores ON and OFF DRT) had a statistically significant effect on muscle responses whatever the emotion or the period of stimulus exposure at the adjusted significance threshold for multiple comparisons (3 muscles x 3 emotions x 4 periods, adjusted *α* value = 0.001). The effects obtained at the significant threshold of 0.05 are nonetheless shown in S3 Appendix.

### Facial reactions and emotion decoding accuracy

At the adjusted significance threshold for multiple comparisons (3 emotions x 4 periods, adjusted *α* value = 0.004), only corrugator responses had a statistically (or quasi-) significant effect on decoding accuracy of expressions of joy: in both groups, for all the 500 ms recording periods except the first, the probability of accurately identifying joy increased with the corrugator relaxation (500–1000: *χ²* = 8.14, *df* = 1, *p* = 0.004; 1000–1500: *χ²* = 9.21, *df* = 1, *p* = 0.002 and 1500–2000: *χ²* = 7.5, *df* = 1, *p* = 0.006). At this threshold, we still noted a statistically marginal group x zygomaticus muscle responses interaction in the joy condition in the first 500 ms period (*χ²* = 5.84, *df* = 1, *p* = 0.016): unlike what was observed in the patients, the probability of accurately identifying joy appeared to increase with the contractions of the zygomaticus muscle in the first 500 ms of stimulus exposure in the HC. The effects associated with a significance threshold of 0.05 are nonetheless shown in S4 Appendix.

## Discussion

The current study was designed to evaluate the role of facial mimicry in recognition of facial emotion. For this purpose, we investigated for the first time EMG responses to facial expressions among patients suffering from Parkinson’s disease (PD) in a facial emotion recognition paradigm. Three main results emerged from our analyses. Firstly, in accordance with the literature, the PD patients were less accurate in decoding facial expressions of joy and neutral faces compared to the healthy controls (HC). Secondly, the facial expressions were mimicked as expected. However, the emotion-specific EMG variations were disturbed in the PD patients with weaker than normal corrugator reactions in response to angry faces and with almost no reactions from the orbicularis and the zygomaticus muscles in response to happy avatars. Thirdly, the analyses highlighted statistically significant effects of the facial reactions on emotion decoding accuracy. Notably, the corrugator relaxation as well as the zygomatic contraction in response to happy faces is correlated to the decoding of joy.

Many studies have demonstrated the negative impact of PD in the recognition of emotion expressed on faces [42–50] but other studies have failed to confirm these observations [51–54]. The review by Assogna and collaborators [19] and the meta-analysis by Gray and Tickle-Degnen [20] identified different factors to explain this discrepancy: the implementation of small samples, inadequate control for demographic characteristics of the participants, the depression status, the presence of cognitive or visuospatial deficits and the influence of dopamine replacement therapy (DRT). In this study, we used large samples (n = 40 in both groups). We excluded individuals suffering from depression, apathy, cognitive and visuospatial deficits or impairments in facial processing. We also took into account the sociodemographic and neuropsychological characteristics of the participants in the statistical analyses. None of these potential confounding factors had a statistically significant effect except for age. As age had the same negative effect in the two groups and since this effect is reported in the literature [55], we did not give further consideration to this point. Finally, we examined the effect of DRT and other clinical features of the patients on emotion decoding accuracy.

Our results confirmed the negative impact of PD in emotion recognition for happy and neutral faces. Conversely, the PD patients did not differ from the HC in the recognition of anger. Furthermore, the nature of the misidentified expressions was globally similar between the groups. Only quantitative and qualitative differences emerged regarding the recognition of joy and neutral expressions. Whatever the emotion displayed, surprise was the most frequent confounding emotion especially among the PD patients. Then, both the HC and the PD patients confounded anger with other negative emotions and neutral with sadness. When the participants had to recognize joy expressions, surprise was the only source of confusion. Since one can be positively surprised, negatively surprised and even neither positively nor negatively surprised but just astonished, surprise is ambiguous. This confounding emotion highlighted difficulties to recognize emotions among PD patients. This effect is supported by studies which depicted surprise as a source of confusion and showed a specific deficit of surprise recognition among PD patients [23,45]. Likewise, the similar nature of the misidentified expressions between HC and PD patients depicted a normal but a noised process of facial emotions. These findings fit with the presumed role of the basal ganglia-thalamocortical connections underlying emotional processing described by Péron and collaborators [56]. According to the model proposed by these authors, a dysfunction in the pathways involving the basal ganglia as it’s observed in PD could prevent correctly inhibiting the non relevant information and/or correctly activating the relevant information causing the emotional judgements to be disturbed.

The fact that the patients’ abilities to recognize emotions tended to increased with the LEDD is fully consistent with the purported role of dopamine in the perception of emotion [57]. A relationship between DRT and emotion recognition is in accordance with both the amygdala dysfunction hypothesis and the dopamine depletion hypothesis in PD. Experimental data support these explanations: for example, the restoration of amygdala response in PD patients perceiving facial emotions during a dopamine-replete state compare to an hypodopaminergic state [58] and the better performances in recognizing facial emotions in medicated compared to unmedicated PD patients [50].

The absence of a negative impact of PD in the recognition of anger fits the idea of Lawrence and collaborators [48,59] that DRT could mask any deficit present in PD especially for anger recognition. In 2007, these authors assessed performances of emotion recognition in PD patients withdrawn from dopamine replacement therapy and showed indeed an anger-specific deficit of recognition among these patients with a spared recognition of other facial emotions (disgust, fear, sadness, joy and surprise). Likewise, Dujardin and collaborators [45] highlighted a deficit in anger recognition in PD patients who had not yet received any medication. Besides, from a methodological point of view, our study had some characteristics which may play a role in our results. First, we used dynamic expressions in order to provide more ecological stimuli. Considering the importance of dynamic features of the stimuli in the emotion recognition process, the used of static faces could artificially cause a deficit in emotion recognition in PD, or at least widened it [19,60]. Like us, Kan and collaborators [47] did not highlighted a deficit concerning the recognition of anger when exposing participants to dynamic facial expressions and they showed that the performances of the patients were largely lower when they had to recognize sadness, disgust and anger from static stimuli compared to dynamic expressions. At last, another argument to explain this absence of group difference concerning the recognition of anger could also reside in the clinical characteristics of the patients. Indeed, the patients involved in the current study did not present any potential non-motor symptoms (cognitive and visuo-perceptual impairment, face processing deficit, depression or apathy) which could have interfered with the performances on the affective recognition test [19,20].

To explain the deficit of facial emotion recognition in PD, some authors have suggested functional and/or anatomical dysfunctions in brain structures such as the amygdala, the basal ganglia including the ventral striatum, the orbito-frontal cortex and the insula as well as the impairment of dopamine transmission in the mesocorticolimbic pathway [18–20]. However, other authors have argued that PD patients could experience deficits in recognizing emotion because of a reduced ability to mimic the perceived emotion. In fact, the presence of a common neural substrate—as part of the mirror neurons system—underlying the ability to express emotions and to recognize facial emotions expressed by others, suggests that facial amimia could contribute to the deficit in recognition of emotion in PD [21,61].

With regard to the EMG responses, our methodology enabled us to evidence emotion-specific facial reactions 500 ms after stimulus onset as described in the literature: among the HC, facial reactions to angry expressions were characterized by an increased activity of the corrugator muscle—an important muscle for frowning in expressions like anger or sadness—and a slight relaxation of the zygomaticus and the orbicularis—muscles involved in the production of smiling expressions by raising the corners of the mouth and forming “crow’s feet” on the outer corners of a person’s eyes [62]—whereas facial reactions to joy are characterized by the reverse pattern. These variations, which occurred only from the first 500 ms after stimulus onset, have been widely highlighted in previous studies and are considered to reflect facial mimicry [63–69].

We also evidenced significant differences regarding these variations between the HC and the PD patients. The responses of the zygomaticus and the orbicularis muscles did not vary with the emotion among the PD patients, as the activity of these muscles did not increase in response to expressions of joy. In addition, the corrugator relaxation in response to happy faces tended to be less marked among the PD patients than in the HC. In response to angry faces, we still noticed an increased activity of the corrugator muscle in the PD patients but it was less marked than that seen in the HC. Thus, PD seems to impact facial mimicry in different manners, with a relatively preserved facial mimicry of angry faces but a considerable disruption of facial mimicry of happy faces. As this could result in an imbalance in favour of the expression of—or reaction to—negative emotions, this phenomenon could contribute to the fact that people suffering from PD are often described by others (including health professionals) as withdrawn, bored or passive, moody, anxious, unhappy or suspicious [70–72]. It is important to note here that the significantly weaker facial reactions to emotions observed among the PD patients could arise from higher tonic muscle activations at baseline. Indeed, analyses on mean EMG amplitudes measured during the last second before stimulus onset showed that zygomaticus muscle exhibited higher activity at baseline in the PD patients compared to the HC. However, no group difference emerged for the corrugator and the orbicularis. As well, we only recorded muscle activity on the left side on the participants’ face and we could wonder whether the laterality of the motor symptoms could play a role for the diminished EMG activity in response to emotion highlighted among the 17 patients with left-side predominant motor symptoms. Nevertheless, analyses focusing on the relationship between clinical characteristics and facial reactions did not highlight any significant effect of the disease laterality.

In this study, the relative preservation of facial mimicry in response to expressions of anger in PD patients could participate in their abilities to recognize anger as accurately as the healthy participants. This would fit with the assumption of embodied simulation theory asserting that mimicry fosters emotion recognition. According to this assumption, the patients’ performances in recognizing joy were expected to collapse because their ability to mimic happy faces was almost inexistent. However, our results did not support this expectation since the patients' decoding accuracy scores for joy remained relatively high despite the negative impact of PD in recognizing joy expressions. The relationship between facial reactions and joy decoding accuracy shown here could provide some elements for discussion. Indeed, our results suggest that corrugator relaxation in response to expressions of joy after 500 ms from stimulus onset fosters the emotion recognition process among both the HC and the PD patients. Among the HC, the information from proprioceptive feedback induced by zygomaticus contraction in the first 500 ms of the perception of the expression also might contribute to the joy recognition. After these first 500 ms, although they still increased, zygomatic contractions did not further boost accuracy in recognizing joy in the HC. This suggests that among the HC, it could be first the information from the early reactions of the zygomaticus muscle and then, the feedback from corrugator relaxation—requiring a longer time frame—that contribute to the recognition of joy. Among the PD patients, the information coming from the zygomaticus muscle seemed to not foster the joy recognition anymore, but—even if corrugator relaxation tended to be weaker than normal—the feedback from corrugator activity might have been still efficient in supporting the recognition of joy. Thus, the findings about joy recognition and joy mimicry are still in favour of the embodiment simulation theory. Finally, we cannot exclude that the motor command related to the mimicry phenomenon might have also an impact on recognition accuracy. Actually, it has been shown that transcranial magnetic stimulation (TMS) above the somatosensory cortices (S1) and motor region (M1) had an impact on mimicry but only TMS on M1 had a behavioural impact on smile detection [73].

The relationship between facial mimicry and emotion recognition observed in this study fits previous findings reporting a positive effect of facial mimicry on recognition of emotions. However, studies investigating the role of facial mimicry in this process have shown mixed results. Some have found that facial mimicry could be considered as a functional element among emotion-related abilities enabling us to infer the emotional state of our interlocutor. This is the case with studies reporting impairment (or improvement) of emotion recognition when facial mimicry is blocked (or intensified) [9,11,74] as well as among people suffering from the locked-in syndrome which leads to a paralysis of facial movements [75]. Moreover, in the study by Sato and colleagues [76], facial EMG activity predicted the recognition of emotional valence through its influence on the experience of emotional valence in response to dynamic facial expressions and Korb et al. [77] showed that facial mimicry predicted authenticity judgments of smiles. In the same way, Künecke and collaborators [78] evidenced a correlation between corrugator responses to angry, happy and sad faces and accuracy of the perception of these emotions.

Conversely, other authors have suggested that facial mimicry is neither necessary nor linked to the process of recognizing emotion. The study by Bogart and Matsumoto [12] among people with Moebius syndrome is in line with this view. Likewise, Hess and Blairy [16] could not confirm any relationship between mimicry and emotion recognition or emotion contagion while Blairy et al. [15] showed that neither spontaneous nor voluntary mimicry increased accuracy in decoding emotions. They did not find a negative impact of "blocking" mimicry—whereby participants were required to show incompatible facial expressions—on decoding accuracy either.

These discrepancies could result from methodological differences including the methods for measuring mimicry (facial EMG *vs*. Ekman’s Facial Action Coding System) and emotion recognition (categorical accuracy scores *vs*. ratings of emotional valence, single task *vs*. multiple tasks approaches), the characteristics of the stimuli (static *vs*. dynamic, prototypical *vs*. more ambiguous) and the analyses conducted (correlations *vs*. path or mediational analyses). This also underlines the importance of dynamic features in relation to facial expressions (stimuli) as well as the importance of taking into account the dynamic aspect of facial reactions (mimicry) in analyses. Psychological and physiological evidences suggest that facial emotions are perceived and mimicked differently when the stimuli are dynamic as opposed to static expressions. Indeed, using static expressions not only affects ecological validity but also limits our understanding of the role of facial mimicry [60].

It is important to note that the positive effect of DRT on emotion recognition—as well as on facial reactions—could conceal possible role of facial feedback in this process. Further investigations assessing facial mimicry among unmedicated PD patients could clarify this point.

Furthermore, we need to interpret these findings carefully given that compensatory strategies could be used by people suffering from a long-lasting motor impairment and not only in temporary experimental manipulations of muscle activity. Indeed, fMRI studies have shown compensatory cortical mechanisms among PD patients [79] and in *Parkin* mutation carriers showing a stronger than normal activity in the ventrolateral premotor cortex (part of the mirror neurons system) during the execution and the perception of affective facial gestures as well as a slightly reduced ability to recognize facial emotions [80].

To conclude, in their recent review, Hess and Fischer [81] claimed that facial mimicry is not necessary to decode emotions but could facilitate the speed of the process [10,82] or the recognition of emotion when the task is difficult. They further reported that facial mimicry is sensitive to the emotional and social context such as the emotional meaning of the facial display, the identity of the sender or the relationship between the observer and the sender. Thus, they suggested that mimicry could occur when it reinforces social bonds, enhances social coordination and improves the quality of social interactions. Therefore, in the same way as facial amimia could lead to inaccurate impressions and reduce the desire for social interaction [70], we can wonder whether the reduction in facial expression of emotion and facial mimicry observed in PD could in turn disturb the way others interpret the emotions of patients and affect the quality of their interactions in real social contexts.

### Conclusions

To sum up, this is the first study to focus on facial mimicry in PD using EMG recordings in a facial emotion recognition paradigm. Using analyses of the temporal aspects of facial EMG reactions in response to dynamic avatars, we highlighted disturbances in facial mimicry among PD patients. In addition, regarding the beneficial effect of mimicry on emotion decoding accuracy evidenced here, reduced facial mimicry could be a new explanatory factor with regard to emotional disturbances associated with PD, notably regarding to the already known deficits in facial expression decoding in PD, once again confirmed in our study. Finally, we provide additional arguments in favour of embodied simulation theory asserting that mimicry could foster the recognition of emotion.

## Supporting Information

S1 Appendix Detailed description of the procedure and the stimulus material. (DOC)

S2 Appendix Inter-muscle comparisons. (DOC)

S3 Appendix Effects of clinical characteristics of the patients on facial reactions to emotion (*α* = 0.05). (DOC)

S4 Appendix Effect of facial reactions on emotion decoding accuracy (*α* = 0.05). (DOC)

S1 Fig EMG data management. For each trial, the last second before stimulus onset was considered as baseline. Then, to examine the temporal profiles of facial reactions to emotions, the EMG amplitudes were averaged on sequential 100 ms intervals (x 20) of stimulus exposure (top panel A) and expressed as a relative percentage of the mean amplitude from baseline (bottom panel A). To examine the impact of medication therapy and disease severity (disease duration, LEDD, Hoehn and Yahr stages and UPDRS III scores both ON and OFF DRT) on EMG responses and to assess the relationship between emotion recognition and facial reactions, facial EMG responses were calculated as previously on sequential 500 ms periods of stimulus exposure. Four periods were thus considered: 0–500; 500–1000; 1000–1500 and 1500–2000 ms (B).
(TIF)

S1 File Sociodemographic, neuropsychological and clinical characteristics of the participants: dataset. Group = healthy controls (HC) and PD patients (PD); Subject = subject number; Sex = participant’s gender (W = woman and M = man); Age = participant’s age at inclusion; STAI_state = state anxiety score on the State-Trait Anxiety Inventory; STAI_trait = trait anxiety score on the State-Trait Anxiety Inventory; Matrix = standard note on the Matrix Reasoning subtest from the Wechsler Adult Intelligence Scale; Benton = standardized score on the on the Benton unfamiliar-face matching test; VOSP_screeningT = score on the shape detection screening subtest from the Visual and Object Space Perception battery; VOSP_posdiscriT = score on the position discrimination VOSP subtest; VOSP_nblocT = score on the number location VOSP subtest; PD_duration = year of diagnosis; PD_laterality = worst affected side (R = right and L = left); UPDRS3_ON and UPDRS3_OFF = scores on the Unified Parkinson’s Disease Rating Scale motor part under dopamine replacement therapy (ON) and during a temporary withdrawal from treatment (OFF); HY_ON and HY_OFF = stages on the Hoehn and Yahr disability scale under dopamine replacement therapy (ON) and during a temporary withdrawal from treatment (OFF); LARS = score on the Lille Apathy Rating Scale; LEDD = levodopa-equivalent daily dose (mg/day).
(TXT)

S2 File Performances on the facial emotion recognition task: dataset. Group = healthy controls (HC) and PD patients (PD); Subject = subject number; Trial = trial number (e01—e36); Emotion = emotion displayed (Angry, Happy, Neutral); Avatar = identification code of the avatar; Decoding_Accuracy = accurately identified expressions were coded as 1 and misidentified expressions were coded as 0; Response = categorical judgements (emotion recognized by the participants).
(TXT)

S3 File EMG responses to emotion displayed: dataset. Group = healthy controls (HC) and PD patients (PD); Subject = subject number; Trial = trial number (e01—e36); Emotion = emotion displayed (Angry, Happy, Neutral); Avatar = identification code of the avatar; Muscle = recorded muscle (Corru = *corrugator supercilii*, Zygo = *zygomaticus major* and Orbi = *orbicularis oculi*); Interval = sequential recording 100 ms interval (i01—i20); EMG_response = EMG amplitudes averaged across the sequential 100 ms intervals of stimulus exposure and expressed as a relative percentage of the mean amplitude for baseline (%).
(TXT)

S1 Table Characteristics of the patients’ medication. Type: L = patients under L-dopa medication only (levodopa + carbidopa and/or levodopa + benserazide and/or levodopa + carbidopa + entacapone), A = under dopamine agonists only, or L+A = under a combination of L-dopa and dopamine agonists; MAO/COMT: Some patients also took monoamine oxidase (MAO) B and/or catechol-O-methytransferase (COMT) inhibitors; Other(s) = Medication in addition of their dopamine replacement therapy. Specificities: ^1^Patient under rotigotine transdermal patches (2 x 8 mg/24 hours) and receiving under-cutaneous injection of apomorphine (1 x 5 mg/24 hours), ^2 & 5^receiving under-cutaneous injections of apomorphine (22 x 3 mg/24 hours and 53 x 4 mg/24 hours), ^3^taking 0.25 mg of alprazolam /24 hours, ^4^12.5 mg of clozapine + 7 drops of clonazepane (2.5 mg/ml)/24 hours.
(DOC)

S2 Table Inter-emotions comparisons of EMG responses recorded on sequential 100 ms intervals of stimulus exposure in the healthy controls. Test statistics (*χ²*) are shown in brackets. Figures in bold denote statistically significant differences (*p* value<0.05). ns = non statistically significant = *p* value>0.1.
(DOC)

S3 Table Inter-emotions comparisons of EMG responses recorded on sequential 100 ms intervals of stimulus exposure in the PD patients. Test statistics (*χ²*) are shown in brackets. Figures in bold denote statistically significant differences (*p* value<0.05). ns = non statistically significant = *p* value>0.1.
(DOC)

S4 Table Inter-muscles comparisons of the EMG responses recorded on sequential 100 ms intervals of stimulus exposure in the healthy controls. CORRU = *corrugator supercilii*; ZYGO = *zygomaticus major*; ORBI = *orbicularis oculi*. Test statistics (*χ²*) are shown in brackets. Figures in bold denote significant differences (*p* value<0.05). ns = non significant = *p* value>0.1.
(DOC)

S5 Table Inter-muscles comparisons of the EMG responses recorded on sequential 100 ms intervals of stimulus exposure in the PD patients. CORRU = *corrugator supercilii*; ZYGO = *zygomaticus major*; ORBI = *orbicularis oculi*. Test statistics (*χ²*) are shown in brackets. Figures in bold denote significant differences (*p* value<0.05). ns = non significant = *p* value>0.1.
(DOC)
