# Supplementary material for: Does Facial Amimia Impact the Recognition of Facial Emotions? An EMG Study in Parkinson’s Disease
Source: PLoS One. 2016 Jul 28;11(7):e0160329. doi: 10.1371/journal.pone.0160329 (PMC4965153; doi:10.1371/journal.pone.0160329)
Supplement: S1 Appendix — (DOC) [file pone.0160329.s001.doc]

**S1 Appendix. Detailed description of the procedure and the stimulus material.**

Participants were seated comfortably and tested independently in a quiet darkened room with a high-resolution monitor (Prolite® screen, 22 inches, 60 Hz refresh rate, IIyama) at a visual distance of 80 cm. For each trial, a grey fixation cross was presented centrally on a black screen for a mean duration of 1550 ± 360.56 ms (standard deviation) to gain the participant’s attention. Then, the participants were exposed to a dynamic avatar (2000 ms) and asked to assess the emotions portrayed on visual analogue scales. The scales remained on the screen for the time of response which was unlimited although the participants were asked to respond as spontaneously as possible. The participants could move as many cursors as they wanted but were asked to move at least one. After they had chosen their response, the participants clicked the “OK, next!” button. Then, after 1000 ms of inter-stimuli interval, the fixation point appeared again and a new trial started.

During the test, the participants were filmed with a webcam connected to a second computer. Seated in front of this observation monitor enabled online control of the data recording, the experimenter remained quietly in the same room standing behind an opaque screen outside the subjects’ field of vision. Breaks of a few minutes were given after each block but the participants could ask for a longer break. The experiment was computerized with the E-Prime Professional software (build 2.02.10.242).

For every stimulus, videos clips (25 ips, 270 Kbps, 800x1200 pixels) in which the emotional expression unfolded from a neutral state to its peak with emotional onset and apex duration of 1000 ms were generated using FACSGen. FACSGen is a dedicated software for creating dynamic facial expressions on the basis of the Facial Action Coding System. This software integrates the texture details of a real human being such as facial hair and skin pigmentation. We thus ensured greater ecological validity by the dynamism and the realism of the avatars. All emotions portrayed were based on emotion-specific action unit (AU) combinations. For anger: AU4 “brow lowerer” + AU5 “upper lids raiser” + AU7 “lids tightener” + AU24 “lips pressor” + AU38 “nostrils dilator”. For joy: AU12 “lips corner puller” + AU6 “cheeks raiser” + AU25 “lips part” + AU26 “jaw drop”). For neutral: to retain the dynamic properties of the stimuli while as far as possible avoiding potential bias by emotional interpretation, the avatar simply closed his eyes (AU43) after 1000 ms of exposure.
