# Supplementary material for: Does Facial Amimia Impact the Recognition of Facial Emotions? An EMG Study in Parkinson’s Disease
Source: PLoS One. 2016 Jul 28;11(7):e0160329. doi: 10.1371/journal.pone.0160329 (PMC4965153; doi:10.1371/journal.pone.0160329)
Supplement: S2 Appendix — (DOC) [file pone.0160329.s002.doc]

**S2 Appendix. Inter-muscle comparisons.**

Among the healthy controls, in response to angry and neutral avatars, the variations of the corrugator muscle significantly differed from those of the zygomaticus and orbicularis from 400 ms after stimulus onset whereas the zygomaticus responses did not differ from those of the orbicularis either for angry or for neutral avatars. Regarding expressions of joy, significant differences appeared from 500 ms after stimulus onset between the recorded activities of the three muscles with a stronger response for the zygomaticus than for the orbicularis. Among the PD patients, corrugator activity in response to angry avatars significantly differed from that of the orbicularis and the zygomaticus muscle from 600 ms after stimulus onset whereas the responses of these two muscles did not differ significantly. There was no significant difference in this group between the reactions of these three muscles in response to happy avatars whatever the interval. The same applied to neutral avatars except from 900 ms after stimulus onset between corrugator and orbicularis reactions.
