# Supplementary material for: Does Facial Amimia Impact the Recognition of Facial Emotions? An EMG Study in Parkinson’s Disease
Source: PLoS One. 2016 Jul 28;11(7):e0160329. doi: 10.1371/journal.pone.0160329 (PMC4965153; doi:10.1371/journal.pone.0160329)
Supplement: S3 Appendix — (DOC) [file pone.0160329.s003.doc]

**S3 Appendix. Effects of clinical characteristics of the patients on facial reactions to emotion (*α*=0.05).**

At the significance threshold of 0.05, none of the patients’ clinical characteristics (disease duration, the worst affected side, LEDD, Hoehn and Yahr stages and UPDRS III scores ON and OFF DRT) had a significant effect on the variations of zygomaticus muscle activity whatever the emotion or the period of stimulus exposure. However, the LEDD had a significant (or quasi-) effect on the reactions of the orbicularis muscle in the anger for all the recorded periods (0-500: *χ²*=4.92, *df*=1, *p*=0.027; 500-1000: *χ²*=4.52, *df*=1, *p*=0.033; 1000-1500: *χ²*=4.2, *df*=1, *p*=0.04 and 1500-2000: *χ²*=6.36, *df*=1, *p*=0.012) as well as in the neutral condition (0-500: *χ²*=3.67, *df*=1, *p*=0.055; 500-1000: *χ²*=2.64, *df*=1, *p*=0.10; 1000-1500: *χ²*=5.06, *df*=1, *p*=0.025; 1500-2000: *χ²*=3.14, *df*=1, *p*=0.076): higher medication dosages led to more marked relaxation of the muscle. In contrast, the least marked relaxation of the orbicularis in response to angry avatars was observed with the most severe motor symptoms for all the recorded periods except the first (UPDRS III scores OFF DRT effect; 500-1000: *χ²*=6.47, *df*=1, *p*=0.019; 1000-1500: *χ²*=4.2, *df*=1, *p*=0.04 and 1500-2000: *χ²*=3.29, *df*=1, *p*=0.07). Likewise, in its more severe stages (Hoehn and Yarh stages OFF DRT effect), the impact of the disease resulted in less marked corrugator relaxation in response to joy expressions during the first and the last periods (0-500: *χ²*=3.96, *df*=1, *p*=0.047 and 1500-2000: *χ²*=4.15, *df*=1, *p*=0.042) and to neutral expressions during the last period (*χ²*=5.23, *df*=1, *p*=0.022).
