# Supplementary material for: Does Facial Amimia Impact the Recognition of Facial Emotions? An EMG Study in Parkinson’s Disease
Source: PLoS One. 2016 Jul 28;11(7):e0160329. doi: 10.1371/journal.pone.0160329 (PMC4965153; doi:10.1371/journal.pone.0160329)
Supplement: S4 Appendix — (DOC) [file pone.0160329.s004.doc]

**S4 Appendix. Effect of facial reactions on emotion decoding accuracy (*α*=0.05).**

In both groups, for all the 500 ms recording periods except the first, corrugator reactions had a significant effect on decoding accuracy for expressions of joy (500-1000: *χ²*=8.14, *df*=1, *p*=0.004; 1000-1500: *χ²*=9.21, *df*=1, *p*=0.002 and 1500-2000: *χ²*=7.5, *df*=1, *p*=0.006): the probability of accurately identifying joy increased with corrugator relaxation from 500 ms after stimulus onset. The group x zygomaticus response interaction had also a significant effect in the joy condition during the first 500 ms period (*χ²*=5.84, *df*=1, *p*=0.016). Unlike what was observed in the patients, the probability of accurately identifying joy increased with the contractions of the zygomaticus muscle during the first 500 ms of stimulus exposure among the HC. The same result was observed during the third period but it did not reach statistical significance (*χ²*=3.49, *df*=1, *p*=0.062).

In response to neutral avatars, only the reactions of the corrugator muscle had an effect on decoding accuracy performances as reflected by a significant interaction effect with the group in the last period (*χ²*=4.28, *df*=1, *p*=0.039). Among the HC, the probability of accurately identifying neutral expressions decreased with the contractions of the corrugator.

Finally, none of the muscle responses were linked to decoding accuracy of anger except for a marginal effect of corrugator responses during the second period (500-1000: *χ²*=2.87, *df*=1, *p*=0.09): unexpectedly, the probability of accurately identifying anger decreased with the contractions of the corrugator muscle irrespective of the group.
