# Supplementary figures and images for: Does Facial Amimia Impact the Recognition of Facial Emotions? An EMG Study in Parkinson’s Disease
Source: PLoS One. 2016 Jul 28;11(7):e0160329. doi: 10.1371/journal.pone.0160329 (PMC4965153; doi:10.1371/journal.pone.0160329)

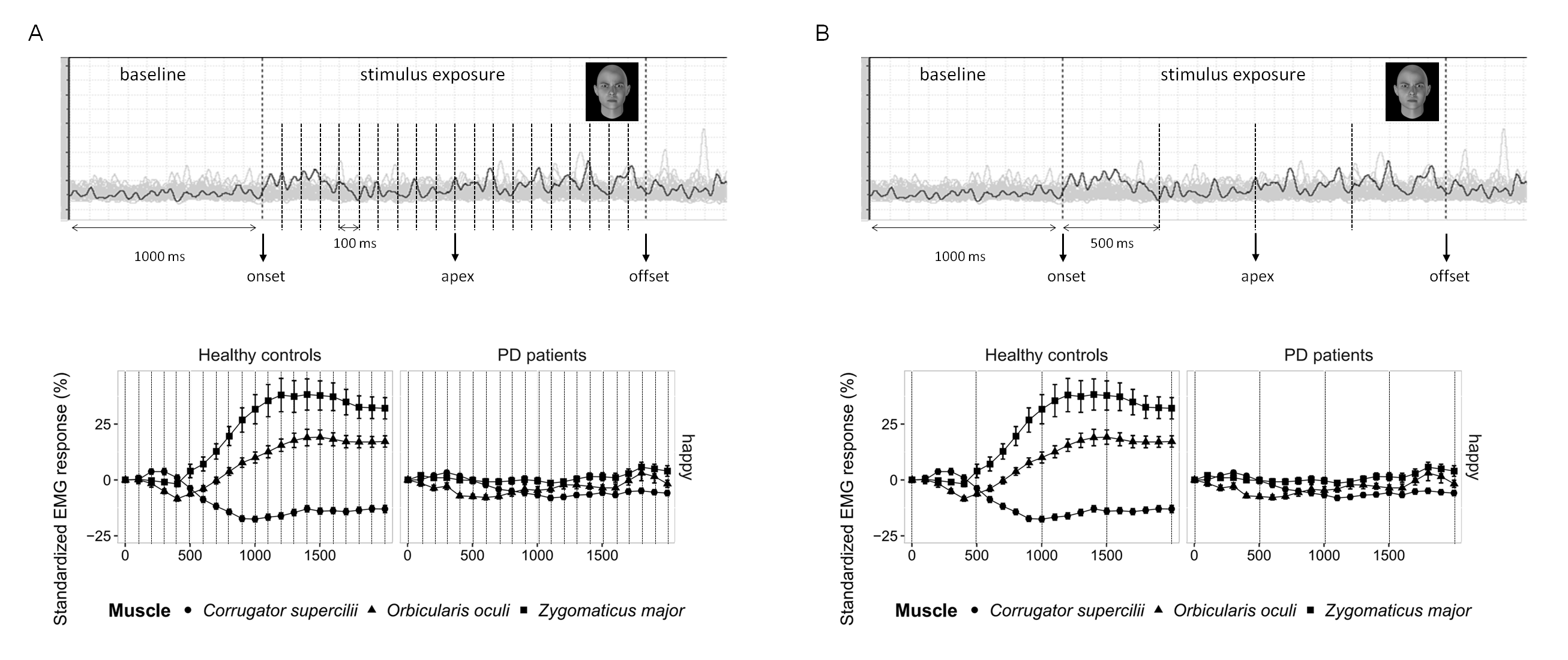

Supplement: S1 Fig — For each trial, the last second before stimulus onset was considered as baseline. Then, to examine the temporal profiles of facial reactions to emotions, the EMG amplitudes were averaged on sequential 100 ms intervals (x 20) of stimulus exposure (top panel A) and expressed as a relative percentage of the mean amplitude from baseline (bottom panel A). To examine the impact of medication therapy and disease severity (disease duration, LEDD, Hoehn and Yahr stages and UPDRS III scores both ON and OFF DRT) on EMG responses and to assess the relationship between emotion recognition and facial reactions, facial EMG responses were calculated as previously on sequential 500 ms periods of stimulus exposure. Four periods were thus considered: 0–500; 500–1000; 1000–1500 and 1500–2000 ms (B). (TIF) [file pone.0160329.s005.tif]
