# Supplementary material for: Does Facial Amimia Impact the Recognition of Facial Emotions? An EMG Study in Parkinson’s Disease
Source: PLoS One. 2016 Jul 28;11(7):e0160329. doi: 10.1371/journal.pone.0160329 (PMC4965153; doi:10.1371/journal.pone.0160329)
Supplement: S1 Table — Type: L = patients under L-dopa medication only (levodopa + carbidopa and/or levodopa + benserazide and/or levodopa + carbidopa + entacapone), A = under dopamine agonists only, or L+A = under a combination of L-dopa and dopamine agonists; MAO/COMT: Some patients also took monoamine oxidase (MAO) B and/or catechol-O-methytransferase (COMT) inhibitors; Other(s) = Medication in addition of their dopamine replacement therapy. Specificities: 1Patient under rotigotine transdermal patches (2 x 8 mg/24 hours) and receiving under-cutaneous injection of apomorphine (1 x 5 mg/24 hours), 2 & 5receiving under-cutaneous injections of apomorphine (22 x 3 mg/24 hours and 53 x 4 mg/24 hours), 3taking 0.25 mg of alprazolam /24 hours, 412.5 mg of clozapine + 7 drops of clonazepane (2.5 mg/ml)/24 hours. (DOC) [file pone.0160329.s009.doc]

S1 Table. Characteristics of the patients’ medication.

| **Patient** | **Type** | **Agonist** | **MAO/COMT** | **Other(s)** | **Patient** | **Type** | **Agonist** | **MAO/COMT** | **Other(s)** |
| --- | --- | --- | --- | --- | --- | --- | --- | --- | --- |
| 1 | L | - | rasagiline | - | 21 | L+A | pramipexole | rasagiline | trihexyphenidyl |
| 2 | L+A | pramipexole | selegiline | amantadine | 22 | L+A | pramipexole | rasagiline | candesartan + propranolol |
| 3 | L+A | pramipexole | - | amantadine | 23 | L+A | pramipexole | selegiline | - |
| 4 | L+A | piribedil | - | - | 24 | L+A | ropinirole | rasagiline | amantadine |
| 5 | L+A | ropinirole | rasagiline | - | 25 | L+A | ropinirole | - | amantadine |
| 6 | A | piribedil | rasagiline | lercanidipine | 26^2^ | L+A | ropinirole | rasagiline | lercanidipine |
| 7 | L | - | - | - | 27 | L | - | rasagiline | trihexyphenidyl |
| 8 | A | pramipexole | rasagiline | - | 28 | L+A | pramipexole | - | - |
| 9 | L+A | pramipexole | rasagiline | - | 293 | L+A | pramipexole | rasagiline | alprazolam |
| 10 | L+A | bromocriptine | rasagiline | - | 30 | L+A | pramipexole | rasagiline | - |
| 11 | L+A | pramipexole | rasagiline | - | 31 | A | pramipexole | rasagiline | acebutolol |
| 12 | L+A | pramipexole | rasagiline | - | 32 | L+A | pramipexole | rasagiline | propranolol + midodrine  + valsartan/hydrochlorothiazide |
| 13 | L+A | pramipexole | rasagiline | metoprolol + rosuvastatine | 33 | L+A | piribedil | rasagiline | midodrine |
| 14 | A | pramipexole | - | - | 34 | L+A | ropinirole | rasagiline | - |
| 15 | L+A | pramipexole | - | trihexyphenidyl | 35 | L+A | ropinirole | rasagiline | - |
| 16 | L+A | pramipexole | rasagiline + entacapone | - | 36^4^ | L | - | rasagiline | amantadine + tamsulosin + clozapine/clonazepane |
| 17 | L+A | pramipexole | rasagiline | - | 37 | L+A | pramipexole | selegiline | - |
| 18 | L | - | - | - | 38 | L+A | ropinirole | rasagiline | amantadine + trihexyphenidyl |
| 19^1^ | L+A | rotigotine | - | irbesartan + hydrochlorothiazide | 39 | L | - | - | - |
| 20 | L+A | ropinirole | rasagiline | - | 40^5^ | L+A | pramipexole | - | bisoprolol |

Type: L = patients under L-dopa medication only (levodopa + carbidopa and/or levodopa + benserazide and/or levodopa + carbidopa + entacapone), A = under dopamine agonists only, or L+A = under a combination of L-dopa and dopamine agonists; MAO/COMT: Some patients also took monoamine oxidase (MAO) B and/or catechol-O-methytransferase (COMT) inhibitors; Other(s) = Medication in addition of their dopamine replacement therapy. Specificities: ^1^Patient under rotigotine transdermal patches (2 x 8 mg/24 hours) and receiving under-cutaneous injection of apomorphine (1 x 5 mg/24 hours), ^2 & 5^receiving under-cutaneous injections of apomorphine (22 x 3 mg/24 hours and 53 x 4 mg/24 hours), ^3^taking 0.25 mg of alprazolam /24 hours, 412.5 mg of clozapine + 7 drops of clonazepane (2.5 mg/ml)/24 hours.
