# Supplementary material for: Does Facial Amimia Impact the Recognition of Facial Emotions? An EMG Study in Parkinson’s Disease
Source: PLoS One. 2016 Jul 28;11(7):e0160329. doi: 10.1371/journal.pone.0160329 (PMC4965153; doi:10.1371/journal.pone.0160329)
Supplement: S2 Table — Test statistics (χ²) are shown in brackets. Figures in bold denote statistically significant differences (p value<0.05). ns = non statistically significant = p value>0.1. (DOC) [file pone.0160329.s010.doc]

S2 Table. Inter-emotions comparisons of EMG responses recorded on sequential 100 ms intervals of stimulus exposure in the healthy controls.

|  | ***Corrugator supercilii*** | | | ***Zygomaticus major*** | | ***Orbicularis oculi*** | |
| --- | --- | --- | --- | --- | --- | --- | --- |
| **Interval** | **happy-angry** | **happy-neutral** | **angry-neutral** | **happy-angry** | **happy-neutral** | **happy-angry** | **happy-neutral** |
| 0-100 | (1.4) ns | (0.23) ns | (2.7) ns | (0.9) ns | (0.3) ns | (1.6) ns | (0.1) ns |
| 100-200 | (2.1) ns | (0.2) ns | (3.6) ns | (0.8) ns | (0.6) ns | (0.3) ns | (1.6) ns |
| 200-300 | (0.01) ns | (1.1) ns | (1.2) ns | (0.3) ns | (0.8) ns | (0.1) ns | (0.1) ns |
| 300-400 | (0.3) ns | (0.1) ns | (0.8) ns | (0) ns | (1.4) ns | (0.4) ns | (0.03) ns |
| 400-500 | **(14.6) <0.05** | (2.7) ns | (4.8) ns | (5) ns | (10.9) ns | (0.2) ns | (1.9) ns |
| 500-600 | **(51.9) <0.001** | (14) =0.066 | (12.1) ns | (8.3) ns | **(19.6) <0.01** | (2.3) ns | (4.8) ns |
| 600-700 | **(94.9) <0.001** | **(30.1) <0.001** | **(18.4) <0.01** | **(27.3) <0.001** | **(41.9) <0.001** | (7) ns | (9.4) ns |
| 700-800 | **(125.6) <0.001** | **(51.8) <0.001** | **(16.3) <0.05** | **(61.4) <0.001** | **(79.3) <0.001** | **(19.5) <0.01** | **(22.8) 0.001** |
| 800-900 | **(145.8) <0.001** | **(78) <0.001** | (10.8) ns | **(116.9) <0.001** | **(126.3) <0.001** | **(35.1) <0.001** | **(36.7) <0.001** |
| 900-1000 | **(152.6) <0.001** | **(91) <0.001** | (8.2) ns | **(160.7) <0.001** | **(168.7) <0.001** | **(51.2) <0.001** | **(52) <0.001** |
| 1000-1100 | **(181) <0.001** | **(92.8) <0.001** | **(14.9) <0.05** | **(1989.3) <0.001** | **(211.5) <0.001** | **(68.2) <0.001** | **(73.9) <0.001** |
| 1100-1200 | **(185) <0.001** | **(75.3) <0.001** | **(24.6) <0.001** | **(222.5) <0.001** | **(225.2) <0.001** | **(76.8) <0.001** | **(84.4) <0.001** |
| 1200-1300 | **(178.7) <0.001** | **(62.5) <0.001** | **(30.2) <0.001** | **(212.3) <0.001** | **(228.3) <0.001** | **(92.6) <0.001** | **(99.4) <0.001** |
| 1300-1400 | **(185.4) <0.001** | **(47.8) <0.001** | **(45.3) <0.001** | **(223.2) <0.001** | **(236.3) <0.001** | **(97.8) <0.001** | **(106.8) <0.001** |
| 1400-1500 | **(175.3) <0.001** | **(52.6) <0.001** | **(36.4) <0.001** | **(218.3) <0.001** | **(232.8) <0.001** | **(91.3) <0.001** | **(113.4) <0.001** |
| 1500-1600 | **(169.1) <0.001** | **(49.3) <0.001** | **(36.2) <0.001** | **(220.3) <0.001** | **(229.6) <0.001** | **(94.3) <0.001** | **(104.9) <0.001** |
| 1600-1700 | **(195.6) <0.001** | **(55.2) <0.001** | **(43.5) <0.001** | **(190.5) <0.001** | **(213.3) <0.001** | **(93.8) <0.001** | **(94.2) <0.001** |
| 1700-1800 | **(213) <0.001** | **(51.5) <0.001** | **(55.5) <0.001** | **(160.3) <0.001** | **(188.5) <0.001** | **(87.4) <0.001** | **(91.3) <0.001** |
| 1800-1900 | **(210.6) <0.001** | **(51.7) <0.001** | **(54.1) <0.001** | **(160.6) <0.001** | **(182.9) <0.001** | **(86.6) <0.001** | **(92.1) <0.001** |
| 1900-2000 | **(229.7) <0.001** | **(48.3) <0.001** | **(97.8) <0.001** | **(166.3) <0.001** | **(183.1) <0.001** | **(88.1) <0.001** | **(92.6) <0.001** |

Test statistics (*χ²*) are shown in brackets. Figures in bold denote statistically significant differences (*p* value<0.05). ns = non statistically significant = *p* value>0.1
