# Supplementary material for: Does Facial Amimia Impact the Recognition of Facial Emotions? An EMG Study in Parkinson’s Disease
Source: PLoS One. 2016 Jul 28;11(7):e0160329. doi: 10.1371/journal.pone.0160329 (PMC4965153; doi:10.1371/journal.pone.0160329)
Supplement: S3 Table — Test statistics (χ²) are shown in brackets. Figures in bold denote statistically significant differences (p value<0.05). ns = non statistically significant = p value>0.1. (DOC) [file pone.0160329.s011.doc]

S3 Table. Inter-emotions comparisons of EMG responses recorded on sequential 100 ms intervals of stimulus exposure in the PD patients.

|  | ***Corrugator supercilii*** | | | ***Zygomaticus major*** | | ***Orbicularis oculi*** | |
| --- | --- | --- | --- | --- | --- | --- | --- |
| **Interval** | **happy-angry** | **happy-neutral** | **angry-neutral** | **happy-angry** | **happy-neutral** | **happy-angry** | **happy-neutral** |
| 0-100 | (0.3) ns | (0.1) ns | (0.03) ns | (0.4) ns | (0.8) ns | (0.8) ns | (0.1) ns |
| 100-200 | (0.2) ns | (0.2) ns | (0) ns | (0.6) ns | (0.5) ns | (0.1) ns | (0.1) ns |
| 200-300 | (0) ns | (0.01) ns | (0) ns | (0.4) ns | (0.1) ns | (0.1) ns | (0.4) ns |
| 300-400 | (0.03) ns | (0.05) ns | (0.2) ns | (0.6) ns | (0.04) ns | (0.3) ns | (0.7) ns |
| 400-500 | (0.6) ns | (1.2) ns | (0.1) ns | (0.7) ns | (0) ns | (0.01) ns | (1.6) ns |
| 500-600 | (5) ns | (3.8) ns | (0.1) ns | (0.3) ns | (0.1) ns | (0.2) ns | (1.9) ns |
| 600-700 | (13.5) =0.086 | (8.6) ns | (0.6) ns | (0.1) ns | (0.6) ns | (0.1) ns | (0.6) ns |
| 700-800 | **(22.4) <0.005** | (10.2) ns | (2.3) ns | (0.1) ns | (0.6) ns | (0.2) ns | (0.04) ns |
| 800-900 | **(25.4) <0.001** | (11.9) ns | (2.6) ns | (0.2) ns | (1) ns | (0.6) ns | (0.1) ns |
| 900-1000 | **(25.9) <0.001** | (14) =0.065 | (1.8) ns | (0.2) ns | (0.6) ns | (0.4) ns | (0.7) ns |
| 1000-1100 | **(31.7) <0.001** | **(23.6) <0.001** | (0.6) ns | (0.1) ns | (0.1) ns | (1.2) ns | (0.9) ns |
| 1100-1200 | **(33) <0.001** | **(21.5) <0.005** | (1.2) ns | (0.4) ns | (0.01) ns | (2.4) ns | (1.7) ns |
| 1200-1300 | **(39.7) <0.001** | **(20.7) <0.005** | (3.1) ns | (1.3) ns | (0.4) ns | (0.9) ns | (2.2) ns |
| 1300-1400 | **(47) <0.001** | **(19.4) <0.005** | (6) ns | (3.4) ns | (0.7) ns | (0.8) ns | (2.1) ns |
| 1400-1500 | **(45.6) <0.001** | **(15.8) <0.05** | (7.7) ns | (3.2) ns | (0.9) ns | (0.4) ns | (1.1) ns |
| 1500-1600 | **(42.7) <0.001** | **(19.4) <0.005** | (4.6) ns | (2.5) ns | (0.9) ns | (0.4) ns | (1.4) ns |
| 1600-1700 | **(45.1) <0.001** | **(14.9) <0.05** | (8.2) ns | (3.3) ns | (1.8) ns | (2.4) ns | (6) ns |
| 1700-1800 | **(51.5) <0.001** | (11.3) ns | **(14.6) <0.05** | (6.2) ns | (4.3) ns | (6.7) ns | (10) ns |
| 1800-1900 | **(59.3) <0.001** | (13.1) =0.105 | **(16.7) <0.05** | (4.6) ns | (3.8) ns | (5.3) ns | (6.6) ns |
| 1900-2000 | **(47.9) <0.001** | **(15.3) <0.05** | (9.1) ns | (3.3) ns | (2.9) ns | (0.5) ns | (2.7) ns |

Test statistics (*χ²*) are shown in brackets. Figures in bold denote statistically significant differences (*p* value<0.05). ns = non statistically significant = *p* value>0.1
