# Supplementary material for: Does Facial Amimia Impact the Recognition of Facial Emotions? An EMG Study in Parkinson’s Disease
Source: PLoS One. 2016 Jul 28;11(7):e0160329. doi: 10.1371/journal.pone.0160329 (PMC4965153; doi:10.1371/journal.pone.0160329)
Supplement: S4 Table — CORRU = corrugator supercilii; ZYGO = zygomaticus major; ORBI = orbicularis oculi. Test statistics (χ²) are shown in brackets. Figures in bold denote significant differences (p value<0.05). ns = non significant = p value>0.1. (DOC) [file pone.0160329.s012.doc]

S4 Table. Inter-muscles comparisons of the EMG responses recorded on sequential 100 ms intervals of stimulus exposure in the healthy controls.

|  | **Angry** | | **Happy** | | | **Neutral** | |
| --- | --- | --- | --- | --- | --- | --- | --- |
| **Interval** | **CORRU-ZYGO** | **CORRU-ORBI** | **CORRU-ZYGO** | **CORRU-ORBI** | **ORBI-ZYGO** | **CORRU-ZYGO** | **CORRU-ORBI** |
| 0-100 | (0.02) ns | (0) ns | (0.01) ns | (0) ns | (0.01) ns | (0) ns | (0.01) ns |
| 100-200 | (3.4) ns | (7.5) ns | (1.9) ns | (3.5) ns | (0.2) ns | (2.9) ns | (7.2) ns |
| 200-300 | (1.3) ns | (8.4) ns | (2.7) ns | (9.6) ns | (2.2) ns | (2.3) ns | (5.7) ns |
| 300-400 | (2.1) ns | (9.8) ns | (0.8) ns | (10.7) ns | (5.6) ns | (2.9) ns | (7.5) ns |
| 400-500 | (11) ns | **(26.6) <0.001** | (7.5) ns | (0.7) ns | (13) ns | (4.8) ns | **(15) <0.05** |
| 500-600 | **(20.2) <0.005** | **(49.2) <0.001** | **(31.3) <0.001** | (2.9) ns | **(15.2) <0.05** | (6.6) ns | **(17.8) <0.01** |
| 600-700 | **(40.6) <0.001** | **(70.5) <0.001** | **(74.3) <0.001** | **(16.2) <0.05** | **(21.2) <0.005** | (11.2) ns | **(20.6) <0.005** |
| 700-800 | **(51.1) <0.001** | **(86.9) <0.001** | **(141.8) <0.001** | **(40) <0.001** | **(31.3) <0.001** | **(17.6) <0.05** | **(31.9) <0.001** |
| 800-900 | **(55.4) <0.001** | **(85.6) <0.001** | **(239) <0.001** | **(76.8) <0.001** | **(45.1) <0.001** | **(21.3) <0.001** | **(37.6) <0.001** |
| 900-1000 | **(61) <0.001** | **(97.7) <0.001** | **(297) <0.001** | **(93) <0.001** | **(57.9) <0.001** | **(28.1) <0.001** | **(50.5) <0.001** |
| 1000-1100 | **(86.2) <0.001** | **(123.3) <0.001** | **(333.6) <0.001** | **(104.6) <0.001** | **(64.9) <0.001** | **(35) <0.001** | **(64) <0.001** |
| 1100-1200 | **(92.1) <0.001** | **(128.3) <0.001** | **(358.6) <0.001** | **(122.2) <0.001** | **(62.5) <0.001** | **(22.6) <0.005** | **(46.4) <0.001** |
| 1200-1300 | **(95) <0.001** | **(136.8) <0.001** | **(331.1) <0.001** | **(127.9) <0.001** | **(47.7) <0.001** | **(23.2) <0.005** | **(43.1) <0.001** |
| 1300-1400 | **(113.4) <0.001** | **(152.8) <0.001** | **(321) <0.001** | **(124.5) <0.001** | **(46) <0.001** | **(19.3) <0.005** | **(37.1) <0.001** |
| 1400-1500 | **(97.7) <0.001** | **(125.8) <0.001** | **(329) <0.001** | **(134.4) <0.001** | **(43.1) <0.001** | **(19) <0.01** | **(39.6) <0.001** |
| 1500-1600 | **(99.5) <0.001** | **(133) <0.001** | **(319.6) <0.001** | **(125.3) <0.001** | **(44.9) <0.001** | **(18.5) <0.01** | **(36.8) <0.001** |
| 1600-1700 | **(112.9) <0.001** | **(162.2) <0.001** | **(295) <0.001** | **(119.9) <0.001** | **(39) <0.001** | **(23.6) <0.001** | **(38.3) <0.001** |
| 1700-1800 | **(123.6) <0.001** | **(176.3) <0.001** | **(261) <0.001** | **(114.1) <0.001** | **(30.2) <0.001** | **(22.3) <0.005** | **(36.6) <0.001** |
| 1800-1900 | **(129) <0.001** | **(178.6) <0.001** | **(251) <0.001** | **(109.7) <0.001** | **(29.1) <0.001** | **(23.7) <0.001** | **(39.9) <0.001** |
| 1900-2000 | **(150.5) <0.001** | **(195.6) <0.001** | **(249.6) <0.001** | **(111.9) <0.001** | **(27.5) <0.001** | **(22) <0.005** | **(36) <0.001** |

CORRU = *corrugator supercilii*; ZYGO = *zygomaticus major*; ORBI = *orbicularis oculi*. Test statistics (*χ²*) are shown in brackets. Figures in bold denote significant differences (*p* value<0.05). ns = non significant = *p* value>0.1
