# Supplementary material for: Does Facial Amimia Impact the Recognition of Facial Emotions? An EMG Study in Parkinson’s Disease
Source: PLoS One. 2016 Jul 28;11(7):e0160329. doi: 10.1371/journal.pone.0160329 (PMC4965153; doi:10.1371/journal.pone.0160329)
Supplement: S5 Table — CORRU = corrugator supercilii; ZYGO = zygomaticus major; ORBI = orbicularis oculi. Test statistics (χ²) are shown in brackets. Figures in bold denote significant differences (p value<0.05). ns = non significant = p value>0.1. (DOC) [file pone.0160329.s013.doc]

S5 Table. Inter-muscles comparisons of the EMG responses recorded on sequential 100 ms intervals of stimulus exposure in the PD patients.

|  | **Angry** | | **Happy** | | | **Neutral** | |
| --- | --- | --- | --- | --- | --- | --- | --- |
| **Interval** | **CORRU-ZYGO** | **CORRU-ORBI** | **CORRU-ZYGO** | **CORRU-ORBI** | **ORBI-ZYGO** | **CORRU-ZYGO** | **CORRU-ORBI** |
| 0-100 | (0.2) ns | (0.03) ns | (0.5) ns | (0.3) ns | (1.5) ns | (0.3) ns | (0.4) ns |
| 100-200 | (2.5) ns | (4.7) ns | (0.1) ns | (3.8) ns | (2.5) ns | (2) ns | (4.2) ns |
| 200-300 | (2) ns | (3.4) ns | (0.6) ns | (4.5) ns | (1.8) ns | (1.2) ns | (2.4) ns |
| 300-400 | (1.6) ns | (5.5) ns | (0.4) ns | (9.6) ns | (6) ns | (1.1) ns | (6) ns |
| 400-500 | (2.5) ns | (9.8) ns | (0.01) ns | (5.9) ns | (6.2) ns | (0.9) ns | (5.1) ns |
| 500-600 | (4.9) ns | (13.6) =0.08 | (0.3) ns | (3.7) ns | (5.9) ns | (2.8) ns | (5.9) ns |
| 600-700 | (7.3) ns | **(19.1) <0.005** | (1.4) ns | (1.2) ns | (5) ns | (6.3) ns | (10.2) ns |
| 700-800 | (11.2) ns | **(28.7) <0.001** | (2.5) ns | (0.1) ns | (3.2) ns | (5.6) ns | (13) ns |
| 800-900 | (11.2) ns | **(26.8) <0.001** | (4.3) ns | (0.3) ns | (2.3) ns | (5.2) ns | (10.1) ns |
| 900-1000 | (10.2) ns | **(24.5) <0.001** | (4.9) ns | (0.5) ns | (2.2) ns | (5) ns | (14.8) ns |
| 1000-1100 | (12.5) ns | **(28) <0.001** | (5.2) ns | (1.8) ns | (0.9) ns | (7.8) ns | (19.2) ns |
| 1100-1200 | **(15.3) <0.05** | **(28.9) <0.001** | (5.6) ns | (3.4) ns | (0.3) ns | (5.4) ns | (16.4) ns |
| 1200-1300 | **(23.3) <0.001** | **(31.6) <0.001** | (6.3) ns | (2.3) ns | (0.9) ns | (6.7) ns | (19.7) ns |
| 1300-1400 | **(33.7) <0.001** | **(40.9) <0.001** | (7.8) ns | (1.5) ns | (2.3) ns | (5.7) ns | (20.9) ns |
| 1400-1500 | **(35.9) <0.001** | **(42.7) <0.001** | (6.1) ns | (0.5) ns | (3) ns | (5.6) ns | (18.1) ns |
| 1500-1600 | **(28.6) <0.001** | **(36.3) <0.001** | (7.2) ns | (1.2) ns | (2.5) ns | (6.8) ns | (19.7) ns |
| 1600-1700 | **(32) <0.001** | **(42.7) <0.001** | (7.7) ns | (2.7) ns | (1.2) ns | (5.7) ns | (21.4) ns |
| 1700-1800 | **(35.2) <0.001** | **(48.6) <0.001** | (13.3) =0.095 | (7.4) ns | (0.8) ns | (3.1) ns | (14.5) ns |
| 1800-1900 | **(37.9) <0.001** | **(56.7) <0.001** | (12.9) =0.12 | (5.7) ns | (1.4) ns | (3.8) ns | (14.3) ns |
| 1900-2000 | **(27.2) <0.001** | **(37.9) <0.001** | (11.7) ns | (1.9) ns | (3.9) ns | (4.7) ns | (17.1) ns |

CORRU = *corrugator* *supercilii*; ZYGO = *zygomaticus* *major*; ORBI = *orbicularis* *oculi*. Test statistics (*χ²*) are shown in brackets. Figures in bold denote significant differences (*p* value<0.05). ns = non significant = *p* value>0.1
